# Automation bias in teachers’ evaluation of student writing: effects of algorithmic warnings and visual risk cues in AI detection reports

**DOI:** 10.3389/fpsyg.2026.1889402

**Published:** 2026-07-07

**Authors:** Peitao Du, Tingting Liu, Xujin Xian

**Affiliations:** 1 Al-Farabi Kazakh National University, Almaty, Kazakhstan; 2 Department of Education, The Catholic University of Korea, Bucheon, Republic of Korea

**Keywords:** AI text detection, algorithmic warning, automation bias, confirmation bias, student writing, teacher evaluation, visual risk cue

## Abstract

As generative artificial intelligence becomes integrated into higher education, teachers increasingly rely on AI text-detection reports to support judgments about authorship, writing quality, and academic integrity. Existing research has mainly examined detector accuracy, false positives, fairness, and policy; less is known about whether report design itself shapes teachers’ evaluations when the judged text is unchanged. This gap matters because numerical scores and visual warnings may frame interpretation, anchor suspicion, and encourage confirmatory reading under uncertainty. Here, we tested how algorithmic warning strength and visual risk cues affect teachers’ evaluations of student writing in a controlled single-stimulus experiment. In a 2 × 2 between-subjects experiment, 214 university teachers evaluated the same medium-quality Chinese social-science course paper accompanied by a fictitious AI-detection report that varied by AI detection rate (7% vs. 87%) and red-highlighting/report-presentation package (absent vs. present). A high detection rate increased perceived AI authorship likelihood and risk and lowered overall quality evaluations, percentage-based scores, originality, language expression, and logical structure. Red highlighting also influenced report perception, language-expression judgments, and self-reported intervention tendency. Significant warning × highlighting interactions emerged for percentage-based scoring, originality, language expression, logical structure, overall multidimensional quality, and intervention tendency, but not for the 1–10 overall rating or report perception. These preliminary and context-specific findings suggest that AI detection reports may function not merely as technical outputs but as socio-technical judgment environments under controlled evaluative conditions. Numerical warnings may anchor teachers’ evaluations, while visual risk cues may selectively amplify suspicion and intervention-oriented responses. Responsible use of AI detection therefore requires neutral report design, independent teacher judgment, human oversight, and training on automation bias.

## 1 Introduction

A single percentage in an artificial intelligence detection report can change how a teacher reads a student paper. As generative artificial intelligence becomes increasingly embedded in higher education, student writing, academic assessment, and academic integrity governance are being reshaped simultaneously. Large language models can produce fluent, coherent, and conventionally structured texts, making it increasingly difficult for teachers to distinguish independent student writing from AI-assisted or largely AI-generated work. In response, universities have turned to AI text detection tools to support assignment review, feedback, and academic integrity decisions. Existing research has discussed both the educational potential of generative AI and its challenges to authorship, originality, and assessment validity (Chan and Hu, 2023; Cotton et al., 2024; Kasneci et al., 2023; Lo, 2023; Perkins, 2023; Tlili et al., 2023). However, the reliability and fairness of AI detection remain contested. Detection systems may produce false positives, vary across linguistic styles, and disadvantage particular groups of writers, including non-native English writers (Elkhatat et al., 2023; Liang et al., 2023; Weber-Wulff et al., 2023). For this reason, scholars have cautioned that detection results should be interpreted alongside academic judgment rather than treated as decisive evidence of misconduct (Chan, 2023; Perkins et al., 2024a; Perkins et al., 2024b).

Yet the key issue for educational assessment is not only whether AI detectors are technically accurate, but also how their reports influence human judgment. AI detection reports are decision-support displays that organize attention and frame interpretation through numerical scores, risk labels, colors, highlighted passages, and explanatory prompts (Goddard et al., 2012; Hoff and Bashir, 2015; Lee and See, 2004; Lyell and Coiera, 2017; Parasuraman and Manzey, 2010; Parasuraman and Riley, 1997; Skitka et al., 2000). A high AI-detection percentage may appear precise and authoritative, while red highlighting may make selected sentences visually salient and easier to interpret as suspicious. Although prior research has examined the accuracy, fairness, and policy legitimacy of AI detection, less is known about how teachers psychologically respond after seeing a detection report. This gap is important because teachers often evaluate student writing under uncertainty: they may not know the student’s writing process, the extent of possible AI assistance, or the reliability of the detection tool. Under such conditions, automated outputs may become judgment anchors (Furnham and Boo, 2011; Tversky and Kahneman, 1974). This process is closely related to automation bias, cognitive anchoring, and confirmation bias (Furnham and Boo, 2011; Goddard et al., 2012; Hoff and Bashir, 2015; Lee and See, 2004; Lyell and Coiera, 2017; Montibeller and von Winterfeldt, 2015; Nickerson, 1998; Parasuraman and Manzey, 2010; Parasuraman and Riley, 1997; Skitka et al., 2000; Tversky and Kahneman, 1974). A numerical warning may first establish a general suspicion frame, while highlighted passages may then provide seemingly concrete evidence that makes the warning easier to accept. In practice, these cues may also operate differently: the detection rate provides a global algorithmic warning about the whole text, whereas sentence-level highlighting provides local evidence targets within the paper.

To address these gaps, this study examines whether algorithmic warning strength and visual risk cues in AI detection reports influence teachers’ evaluations of the same student paper under controlled experimental conditions. Using a 2 × 2 between-subjects experimental design, university teachers were assigned to report conditions varying in AI detection rate and red-highlighting presentation, while the student paper itself was held constant. This design allows the study to test whether differences in evaluation can be attributed to report cues rather than to differences in writing quality. Specifically, the study investigates whether a high AI detection rate lowers academic quality evaluations, whether a red-highlighting/report-presentation package independently shapes judgment, and whether the two cues reinforce each other. It also examines whether these effects vary across dimensions of writing evaluation and whether report cues influence teachers’ self-reported intervention-oriented responses (Furnham and Boo, 2011; Goddard et al., 2012; Hoff and Bashir, 2015; Lee and See, 2004; Lyell and Coiera, 2017; Montibeller and von Winterfeldt, 2015; Nickerson, 1998; Parasuraman and Manzey, 2010; Parasuraman and Riley, 1997; Skitka et al., 2000; Tversky and Kahneman, 1974). By treating AI detection reports as potential socio-technical judgment environments rather than purely technical outputs, this study contributes preliminary evidence to research on AI-assisted assessment, human–automation interaction, and academic integrity governance. It also offers practical implications for the responsible design and use of AI detection tools in higher education (Chan, 2023; Perkins et al., 2024a; Perkins et al., 2024b), while recognizing that generalization beyond the present single-stimulus Chinese-language experiment requires further replication.

## 2 Theoretical background and hypotheses

### 2.1 AI text detection as an educational judgment context

Generative AI has created a new assessment problem in higher education: teachers must evaluate not only the quality of student writing but also the possibility that a text was produced or substantially assisted by a language model. Existing scholarship has discussed the educational opportunities and risks of large language models, including their role in writing support, assessment, academic integrity, and the need for human oversight (Chan and Hu, 2023; Chan, 2023; Cotton et al., 2024; Kasneci et al., 2023; Lo, 2023; Perkins, 2023; Tlili et al., 2023). In response, AI text detection tools have increasingly been positioned as auxiliary instruments for identifying possible AI-generated content (Elkhatat et al., 2023; Perkins et al., 2024a; Perkins et al., 2024b; Weber-Wulff et al., 2023).

However, the literature also shows that AI detection is not a neutral or fully reliable solution. Detectors may produce false positives, may be sensitive to linguistic style, and may disadvantage particular groups of writers. For example, research has shown that some GPT detectors misclassify non-native English writing as AI-generated at disproportionately high rates (Liang et al., 2023). Other work on higher education assessment has emphasized that detection software should be combined with academic judgment rather than treated as decisive evidence (Perkins et al., 2024a; Perkins et al., 2024b). These studies have clarified important questions about accuracy, fairness, and policy legitimacy (Chan, 2023; Elkhatat et al., 2023; Perkins et al., 2024a; Perkins et al., 2024b; Weber-Wulff et al., 2023).

Less is known, however, about the psychological effect of detection reports on teachers who read them. A report does not simply state a technical result; it presents numbers, labels, colors, and highlighted passages that may change the way evaluators interpret the same paper (Furnham and Boo, 2011; Goddard et al., 2012; Hoff and Bashir, 2015; Lee and See, 2004; Lyell and Coiera, 2017; Montibeller and von Winterfeldt, 2015; Nickerson, 1998; Parasuraman and Manzey, 2010; Parasuraman and Riley, 1997; Skitka et al., 2000; Tversky and Kahneman, 1974). The present study therefore treats the AI detection report as a decision-support display rather than as a purely technical output (Goddard et al., 2012; Hoff and Bashir, 2015; Lee and See, 2004; Lyell and Coiera, 2017; Parasuraman and Manzey, 2010; Parasuraman and Riley, 1997; Skitka et al., 2000). The central issue is whether algorithmic warning strength and visual risk cues alter teachers’ quality evaluations and intervention-oriented responses when the student paper itself is held constant.

### 2.2 Automation bias and algorithmic warning strength

Automation bias refers to the tendency to over-rely on automated systems or decision-support tools, especially under uncertainty (Goddard et al., 2012; Lyell and Coiera, 2017; Parasuraman and Manzey, 2010; Parasuraman and Riley, 1997; Skitka et al., 2000). Classic work on human-automation interaction distinguishes appropriate use from misuse, including cases in which users defer excessively to automated advice (Parasuraman and Manzey, 2010; Parasuraman and Riley, 1997). Related research shows that automated aids can reduce independent verification and increase omission or commission errors when decision makers treat system outputs as more authoritative than other available evidence (Goddard et al., 2012; Lyell and Coiera, 2017; Skitka et al., 2000).

AI text detection creates a similar judgment context. Teachers often cannot directly observe a student’s writing process and must infer authorship from textual quality, writing style, and available contextual information. A numerical output such as “AI content: 87%” appears precise and technologically authoritative, even when users know that detection tools can be fallible (Elkhatat et al., 2023; Liang et al., 2023; Perkins et al., 2024a; Perkins et al., 2024b; Weber-Wulff et al., 2023). The number may therefore serve as an algorithmic warning that anchors the evaluator’s judgment before the paper is assessed in detail, consistent with classic and later work on anchoring heuristics under uncertainty (Furnham and Boo, 2011; Kahneman, 2003; Tversky and Kahneman, 1974).

In this study, algorithmic warning strength is operationalized as a low AI detection rate (7%) versus a high AI detection rate (87%). If teachers rely on the detection percentage as a strong external cue, the high-rate condition should increase perceived AI authorship and lower evaluations of the same paper’s academic quality (Araujo et al., 2020; Burton et al., 2020; Dietvorst et al., 2015; Furnham and Boo, 2011; Goddard et al., 2012; Hoff and Bashir, 2015; Kahneman, 2003; Lee and See, 2004; Logg et al., 2019; Lyell and Coiera, 2017; Parasuraman and Manzey, 2010; Parasuraman and Riley, 1997; Skitka et al., 2000; Tversky and Kahneman, 1974).

Therefore, this study proposes:

*H1*: Compared with teachers in the low-AI-detection-rate condition, teachers in the high-AI-detection-rate condition will give the same student paper a lower overall academic quality evaluation (Furnham and Boo, 2011; Goddard et al., 2012; Hoff and Bashir, 2015; Kahneman, 2003; Lee and See, 2004; Lyell and Coiera, 2017; Parasuraman and Manzey, 2010; Parasuraman and Riley, 1997; Skitka et al., 2000; Tversky and Kahneman, 1974).

### 2.3 Visual risk cues, attention guidance, and cognitive anchoring

Detection reports do not rely only on numerical scores. They also use interface cues that direct attention and frame interpretation. Color is one such cue: research on color psychology suggests that color can carry contextual meanings and influence cognition and behavior (Elliot and Maier, 2007; Elliot and Maier, 2014). Red is especially relevant in evaluative interfaces because it is often associated with errors, danger, warning, or the need for corrective action (Braun and Silver, 1995; Kuniecki et al., 2015; Pravossoudovitch et al., 2014). Experimental evidence also suggests that red can attract attention in emotionally meaningful contexts (Kuniecki et al., 2015; Pravossoudovitch et al., 2014).

In an AI detection report, red highlighting may therefore work as more than a formatting choice. It can mark selected sentences as visually salient and guide teachers toward specific parts of the paper (Braun and Silver, 1995; Elliot and Maier, 2007; Elliot and Maier, 2014; Kuniecki et al., 2015; Pravossoudovitch et al., 2014). Ordinary formulaic expressions, such as introductory or concluding phrases, may be interpreted differently once they are displayed as highlighted risk evidence. A sentence that would otherwise appear conventional may become an apparent sign of AI generation when visually framed by the detection report (Barkaoui, 2007; Deane, 2013; Furnham and Boo, 2011; Lim, 2011; Montibeller and von Winterfeldt, 2015; Nickerson, 1998; Ramesh and Sanampudi, 2022; Tversky and Kahneman, 1974).

In this study, the visual risk cue is treated as a red-highlighting report-presentation package rather than as a pure color-only manipulation. It includes sentence-level red highlighting and condition-consistent wording that frames selected passages as requiring manual review. This operational definition is important because the manipulation combines visual salience with report-based interpretive guidance (Braun and Silver, 1995; Elliot and Maier, 2007; Elliot and Maier, 2014; Kuniecki et al., 2015; Pravossoudovitch et al., 2014).

Therefore, this study proposes:

*H2*: Compared with teachers in the no-red-highlighting condition, teachers in the red-highlighting condition (referring to the full report-presentation package defined above) will give the same student paper a lower academic quality evaluation (Barkaoui, 2007; Braun and Silver, 1995; Deane, 2013; Elliot and Maier, 2007; Elliot and Maier, 2014; Kuniecki et al., 2015; Lim, 2011; Pravossoudovitch et al., 2014; Ramesh and Sanampudi, 2022).

### 2.4 Joint effects of algorithmic warnings and visual evidence cues

Algorithmic warning strength and visual risk cues may also reinforce each other. The AI detection rate provides a global judgment frame: it tells the teacher whether the system views the whole text as low risk or high risk. The red-highlighting/report-presentation condition provides local evidence targets: it directs the teacher toward particular sentences that appear to support the system’s conclusion. The two cues may therefore operate through different but complementary pathways (Araujo et al., 2020; Burton et al., 2020; Dietvorst et al., 2015; Furnham and Boo, 2011; Goddard et al., 2012; Hoff and Bashir, 2015; Kahneman, 2003; Lee and See, 2004; Logg et al., 2019; Lyell and Coiera, 2017; Montibeller and von Winterfeldt, 2015; Nickerson, 1998; Parasuraman and Manzey, 2010; Parasuraman and Riley, 1997; Skitka et al., 2000; Tversky and Kahneman, 1974).

This distinction is important because a high AI rate without highlighting provides a conclusion but little visible textual evidence, whereas red highlighting without a high AI rate provides local salience but a weaker overall suspicion frame. When both cues are present, teachers may see the high percentage as the system’s general judgment and the highlighted passages as concrete evidence. In other words, the numerical warning may first establish a general suspicion frame, while highlighted passages may then provide seemingly concrete evidence that makes the warning easier to accept. This combination may encourage confirmatory reading and make the negative evaluation of the paper more likely (Araujo et al., 2020; Burton et al., 2020; Dietvorst et al., 2015; Furnham and Boo, 2011; Kahneman, 2003; Logg et al., 2019; Montibeller and von Winterfeldt, 2015; Nickerson, 1998; Tversky and Kahneman, 1974).

Therefore, this study proposes:

*H3*: There will be an interaction between algorithmic warning and visual risk cue. The negative effect of a high AI detection rate on teachers’ evaluations will be stronger when red highlighting is present (Araujo et al., 2020; Burton et al., 2020; Dietvorst et al., 2015; Furnham and Boo, 2011; Goddard et al., 2012; Hoff and Bashir, 2015; Kahneman, 2003; Lee and See, 2004; Logg et al., 2019; Lyell and Coiera, 2017; Montibeller and von Winterfeldt, 2015; Nickerson, 1998; Parasuraman and Manzey, 2010; Parasuraman and Riley, 1997; Skitka et al., 2000; Tversky and Kahneman, 1974).

### 2.5 Confirmation bias and dimensional differences in writing evaluation

Confirmation bias refers to the tendency to seek, interpret, or remember information in ways that support an existing belief or hypothesis (Montibeller and von Winterfeldt, 2015; Nickerson, 1998). In the present study, a high AI detection rate may first create the hypothesis that the paper is likely AI-assisted. The red-highlighting condition may then guide teachers toward sentence-level evidence that appears to confirm this suspicion (Braun and Silver, 1995; Elliot and Maier, 2007; Elliot and Maier, 2014; Furnham and Boo, 2011; Kuniecki et al., 2015; Montibeller and von Winterfeldt, 2015; Nickerson, 1998; Pravossoudovitch et al., 2014; Tversky and Kahneman, 1974). As a result, teachers may reinterpret ordinary features of student writing as signs of AI generation (Barkaoui, 2007; Deane, 2013; Lim, 2011; Ramesh and Sanampudi, 2022).

The effect should not necessarily be uniform across all dimensions of writing evaluation. Language expression is the most directly observable dimension and is closely tied to the highlighted sentences. Formulaic wording, fluent but generic phrasing, and conventional transitions are likely to become salient once a report encourages suspicion (Barkaoui, 2007; Braun and Silver, 1995; Deane, 2013; Elliot and Maier, 2007; Elliot and Maier, 2014; Kuniecki et al., 2015; Lim, 2011; Pravossoudovitch et al., 2014; Ramesh and Sanampudi, 2022). Originality may also be affected, because perceived AI authorship can imply reduced independent thinking. Logical structure, however, is a deeper argumentative feature and may be less immediately tied to sentence-level highlighting, although it can still decline if teachers generalize suspicion from local language cues to the whole paper (Deane, 2013; Montibeller and von Winterfeldt, 2015; Nickerson, 1998; Ramesh and Sanampudi, 2022).

Therefore, this study proposes:

*H4*: Visual risk cues will show a descriptively stronger effect on language-expression evaluations than on deeper dimensions such as logical structure, while originality evaluations may also decline when teachers infer reduced independent authorship. Because this hypothesis concerns the relative pattern across evaluation dimensions, it is treated as descriptive unless effect-size differences are tested directly (Barkaoui, 2007; Deane, 2013; Lakens, 2013; Lim, 2011; Ramesh and Sanampudi, 2022; Richardson, 2011).

### 2.6 From evaluative judgment to intervention-oriented responses

If AI detection reports shape evaluative judgment, their influence may extend beyond scores. In educational settings, suspicion of AI-assisted writing can lead teachers to request revisions, remind students about writing independence or AI-use norms, or ask students to explain their writing process and sources. These responses are not identical to formal penalties, but they still matter because they can affect feedback, student-teacher interaction, and the governance of academic integrity (Chan, 2023; Cotton et al., 2024; Perkins et al., 2024a; Perkins et al., 2024b; Perkins, 2023).

The present study therefore distinguishes academic-quality evaluation from self-reported intervention-oriented responses. This distinction keeps the behavioral claim cautious: the experiment measures teachers’ stated tendencies after reading the paper and report, not actual grading behavior or disciplinary action. Nevertheless, if warning strength and visual cues increase these tendencies, it would suggest that detection reports can influence both cognitive evaluation and anticipated pedagogical response (Goddard et al., 2012; Hoff and Bashir, 2015; Lee and See, 2004; Lyell and Coiera, 2017; Montibeller and von Winterfeldt, 2015; Nickerson, 1998; Parasuraman and Manzey, 2010; Parasuraman and Riley, 1997; Skitka et al., 2000).

Therefore, this study proposes:

*H5*: High AI detection rates and the red-highlighting condition (again referring to the report-presentation package) will increase teachers’ self-reported intervention-oriented responses, including requiring revision, reminding students about writing independence or AI-use norms, and requesting explanations of the writing process (Chan, 2023; Cotton et al., 2024; Goddard et al., 2012; Hoff and Bashir, 2015; Lee and See, 2004; Lyell and Coiera, 2017; Parasuraman and Manzey, 2010; Parasuraman and Riley, 1997; Perkins et al., 2024a; Perkins et al., 2024b; Perkins, 2023; Skitka et al., 2000).

## 3 Methods

### 3.1 Experimental design

This study used a 2 × 2 between-subjects design. The first independent variable was algorithmic warning strength, operationalized as a low AI detection rate (7%) versus a high AI detection rate (87%). The second independent variable was visual risk cue, operationalized as the absence versus presence of a red-highlighting/report-presentation package. The four conditions were Group A (low/no highlighting), Group B (low/red), Group C (high/no highlighting), and Group D (high/red). Factorial between-subjects designs are appropriate when researchers need to estimate main effects and interactions while preserving the temporal priority of experimentally assigned manipulations (Lakens, 2013; Richardson, 2011; Simmons et al., 2011).

All participants read the same student course paper; only the accompanying AI detection report differed. This design allowed differences in evaluation to be attributed to report cues while holding paper quality constant. The 7%-vs.-87% contrast was intentionally strong so that the study could test whether teachers’ judgments are sensitive to clearly low versus clearly high algorithmic warnings; generalization to real-world intermediate detection rates is considered cautiously in the Discussion. The four conditions are summarized in Supplementary Table B1, and a visual overview of the experimental design is provided in Supplementary Figure G1 (Furnham and Boo, 2011; Goddard et al., 2012; Hoff and Bashir, 2015; Lee and See, 2004; Lyell and Coiera, 2017; Montibeller and von Winterfeldt, 2015; Nickerson, 1998; Parasuraman and Manzey, 2010; Parasuraman and Riley, 1997; Simmons et al., 2011; Skitka et al., 2000; Tversky and Kahneman, 1974).

### 3.2 Participants

University teachers with experience grading papers, assignments, or related teaching tasks were recruited through Wenjuanxing and teacher communities. Recruitment was not limited to one university or region. Participation was voluntary and anonymous, with a small platform-based payment. Of 232 initial responses, 18 were removed according to prespecified cleaning rules, leaving 214 valid participants: Group A = 53, Group B = 52, Group C = 55, and Group D = 54. All valid participants passed the attention check and reported relevant grading experience. Detailed sample flow is shown in Supplementary Table G1. Online survey experiments commonly use attention checks and data-screening procedures to reduce inattentive or careless responding (Berinsky et al., 2014; Curran, 2016; DeSimone et al., 2015; Krosnick, 1991; Meade and Craig, 2012).

In the valid sample, social sciences were the largest field (144, 67.3%), followed by science and engineering (45, 21.0%), humanities (21, 9.8%), and medicine (4, 1.9%). Teaching experience was mainly 4–10 years (147, 68.7%), followed by 11–20 years (37, 17.3%) and 1–3 years (30, 14.0%); no participant reported more than 20 years. Sixteen participants (7.5%) had used AI text detection or AI plagiarism-checking tools, and all had experience grading course papers, theses, or assignments. These background variables were retained for descriptive reporting and balance checks because prior experience and domain context can shape reliance on automated judgment aids (Araujo et al., 2020; Burton et al., 2020; Dietvorst et al., 2015; Hoff and Bashir, 2015; Lee and See, 2004; Logg et al., 2019).

### 3.3 Experimental materials

#### 3.3.1 Student course paper

The experimental material was a Chinese course paper, “The Effects and Challenges of Online Education.” It was constructed as a medium-quality undergraduate-style paper with a basic structure and fluent language, but conventional viewpoints and limited argumentative depth. This level of quality was chosen to avoid floor or ceiling effects that might mask or exaggerate cue effects. However, the medium-quality level was researcher-constructed rather than independently validated through a pilot study or external rater calibration, so this stimulus-quality assumption should be interpreted cautiously and is acknowledged as a limitation. The paper covered introduction, learning effects, interaction modes, technical conditions, and conclusion, allowing teachers from different disciplines to evaluate it. The material was administered in Chinese; Supplementary Appendix A provides an English translation for review purposes, and the original Chinese stimulus materials can be supplied as Supplementary material if required by the journal. Writing-evaluation research shows that raters respond to scale structure, language features, and argumentation-related constructs, which supports the choice of a medium-quality text rather than an obviously excellent or poor paper (Barkaoui, 2007; Deane, 2013; Lim, 2011; Ramesh and Sanampudi, 2022).

Four formulaic target sentences were embedded to support the visual-risk-cue manipulation. These sentences contained no obvious factual errors or logical breaks, but their wording was conventional enough to be read as formulaic. The paper content was identical across groups; only the target sentences appeared in red in the red-highlighting conditions and in black in the no-highlighting conditions. The full paper is provided in Supplementary Appendix A. This manipulation is consistent with research showing that visually salient color cues and warning formats can alter perceived risk and attention allocation (Braun and Silver, 1995; Elliot and Maier, 2007; Elliot and Maier, 2014; Kuniecki et al., 2015; Pravossoudovitch et al., 2014).

#### 3.3.2 AI detection report

A fictitious system, “AcademicCheck,” was used to create the AI writing analysis reports, thereby reducing possible confounding from participants’ prior attitudes toward real tools. The four reports were identical in system name, layout, title, submission time, word count, language, review mode, and basic information. The substantive manipulated cues were the AI detection rate and the report-presentation package, which included sentence-level red highlighting and condition-specific prompt wording. This design focused on decision-support cues that have been linked to automation bias and trust calibration (Araujo et al., 2020; Burton et al., 2020; Dietvorst et al., 2015; Goddard et al., 2012; Hoff and Bashir, 2015; Lee and See, 2004; Logg et al., 2019; Lyell and Coiera, 2017; Parasuraman and Manzey, 2010; Parasuraman and Riley, 1997; Skitka et al., 2000).

The visual risk cue should therefore be interpreted as a report-presentation package rather than as a pure color-only manipulation. It combined sentence-level red highlighting with prompt wording that was coherent with the assigned detection-rate and highlighting condition (see Supplementary Table B1). Hereafter, the terms red-highlighting condition and visual-risk-cue condition refer to this package unless otherwise specified (Braun and Silver, 1995; Elliot and Maier, 2007; Elliot and Maier, 2014; Kuniecki et al., 2015; Pravossoudovitch et al., 2014).

Group A showed 7% AI content with no red highlighting; Group B showed 7% with the four target sentences highlighted; Group C showed 87% with no highlighting; and Group D showed 87% with the four target sentences highlighted. Detailed report prompts and condition descriptions are provided in Supplementary Table B1. The strong cue contrast was suitable for testing sensitivity to warning strength, although the expected effect size should be interpreted cautiously when generalizing to ordinary teaching contexts (Furnham and Boo, 2011; Goddard et al., 2012; Hoff and Bashir, 2015; Lakens, 2013; Lee and See, 2004; Lyell and Coiera, 2017; Parasuraman and Manzey, 2010; Parasuraman and Riley, 1997; Richardson, 2011; Skitka et al., 2000; Tversky and Kahneman, 1974).

### 3.4 Measures

The questionnaire and scales were finalized before administration. All groups received the same items, item order, and response ranges; only the stimulus materials differed. Standardized measurement across conditions helps ensure that post-treatment differences can be attributed to the manipulated stimuli rather than to item-format variation (Curran, 2016; DeSimone et al., 2015; Krosnick, 1991; Meade and Craig, 2012; Norman, 2010; Simmons et al., 2011).

#### 3.4.1 Overall academic quality evaluation

Overall academic quality was measured with two items: a 1–10 overall quality rating (1 = very poor, 10 = excellent) and a 0–100 course paper score. Using both a holistic rating and a finer percentage-based score allowed the analysis to examine whether scale granularity affected sensitivity to experimental cues (Barkaoui, 2007; Lakens, 2013; Norman, 2010; Richardson, 2011).

#### 3.4.2 Multidimensional academic quality evaluation

Multidimensional academic quality was measured on a 7-point Likert scale (1 = strongly disagree, 7 = strongly agree) across three dimensions: originality, language expression, and logical structure. Each dimension included three items: independent viewpoints, independent thinking, and novelty for originality; fluency, course-paper conventions, and natural writing for language expression; and clear argumentative structure, section coherence, and support for main arguments for logical structure. Likert-type scales and analytic writing-evaluation dimensions are widely used in educational and psychological measurement, but their reliability and construct breadth should be assessed rather than assumed (Barkaoui, 2007; Cronbach, 1951; Deane, 2013; Lim, 2011; McNeish, 2018; Norman, 2010; Ramesh and Sanampudi, 2022; Tavakol and Dennick, 2011).

#### 3.4.3 Behavioral intervention tendency

Behavioral intervention tendency was measured on a 7-point scale (1 = definitely would not, 7 = definitely would). The three items asked whether participants would require revision, remind the student about writing independence or AI-use norms, and request explanations of the writing process or sources. Because these behaviors all reflect further intervention, they were combined into a composite index, with the one-factor structure examined in the reliability analysis. Internal-consistency reporting is appropriate for such multi-item indices, although very high alpha values should still be interpreted with attention to item redundancy and construct coverage (Cronbach, 1951; McNeish, 2018; Tavakol and Dennick, 2011).

#### 3.4.4 Manipulation checks

Manipulation checks covered perceived likelihood that the paper was generated or assisted by AI, perceived AI risk level shown by the report, and recall of the AI content value. The recall item confirmed whether participants noticed the key manipulation. Manipulation checks and attention/recall items are commonly used to evaluate whether experimental stimuli were perceived as intended and to diagnose inattentive responding (Berinsky et al., 2014; Curran, 2016; DeSimone et al., 2015; Krosnick, 1991; Meade and Craig, 2012).

#### 3.4.5 AI detection report perception

The AI detection report perception scale used a 7-point Likert format to assess perceived informational value, influence on source judgment, attention to formulaic expressions, use of risk cues, and independent judgment. The independent-judgment item was reverse scored and averaged with the other items. Coding procedures are reported in Supplementary Table D1, and the full scale appears in Supplementary Appendix C. Reverse coding and composite-score construction require reliability checking and transparent documentation of scoring rules (Cronbach, 1951; McNeish, 2018; Norman, 2010; Tavakol and Dennick, 2011).

### 3.5 Procedure

Participants entered through the questionnaire link, read informed consent, and were randomly assigned by Wenjuanxing to one of four conditions. They first viewed the corresponding AcademicCheck report summary and AI content cue, and then read the same student paper. In the red-highlighting conditions, four target sentences appeared in red. Participants then completed the overall quality evaluation, multidimensional quality evaluation, intervention-tendency items, manipulation checks, report-perception items, open-ended suggestions, suspicion probe, and demographic questions. This sequence preserved the order from stimulus exposure to outcome measurement and supported internal validity in cue-effect testing (Berinsky et al., 2014; Curran, 2016; DeSimone et al., 2015; Krosnick, 1991; Meade and Craig, 2012; Simmons et al., 2011).

To improve data quality, the questionnaire included an attention check, detection-rate recall item, open-ended suggestion, and suspicion probe. These indicators were used in combination rather than as a single exclusion rule, consistent with recommendations for screening online survey data (Berinsky et al., 2014; Curran, 2016; DeSimone et al., 2015; Krosnick, 1991; Meade and Craig, 2012).

### 3.6 Ethical considerations

This study received ethical approval from the Asia-Pacific Think Tank Research Institute Institutional Review Board (Approval No.: APTTRI-IRB-2026-905386; approval date: February 25, 2026). The review determined that the project did not involve animal experiments, clinical trials, biomedical intervention, biological specimens, minors, patients, or identifiable student academic records, and that it constituted a minimal-risk social-science study using simulated materials and questionnaire measures. All participants were adult university teachers who participated voluntarily after reading online informed consent. The consent information explained the study purpose, procedures, voluntary nature of participation, right to withdraw, data use, and privacy protection. Responses were analyzed only in aggregate form; personal identifiers were not reported; and the data were stored securely and accessed only by the research team. Because the AI detection reports and student paper were fictitious research materials, participants were debriefed after completing the questionnaire that the AI detection report was simulated and did not constitute a real academic-integrity judgment.

### 3.7 Data analysis strategy

Responses were first screened using prespecified quality-control criteria, including attention-check failure, abnormal completion time combined with other invalid-response indicators, invariant scale responses, clear misidentification of the manipulated AI-rate cue, contradictory manipulation-check responses, explicit reconstruction of the experimental purpose, and low-quality open-ended responses as an auxiliary indicator. Ambiguous recall responses were retained only when other manipulation-check indicators were consistent with the assigned condition and when no additional invalid-response indicator was present. The recall-coding criteria, ambiguous-case handling rule, and exclusion logic are documented in Supplementary Table D1; recall-check error cases without other exclusion reasons were also reintroduced in a robustness check to evaluate whether the main conclusions depended on this decision (Berinsky et al., 2014; Curran, 2016; DeSimone et al., 2015; Krosnick, 1991; Meade and Craig, 2012).

Internal consistency was then assessed for all multi-item scales (Cronbach, 1951; McNeish, 2018; Tavakol and Dennick, 2011). The primary inferential analyses used 2 × 2 factorial ANOVAs to test the effects of algorithmic warning strength, visual risk cue, and their interaction on manipulation checks and dependent variables. Because multiple related dependent variables were tested, uncorrected p values are reported for transparency, but the interpretation of the primary ANOVA results also considers Benjamini–Hochberg false-discovery-rate (FDR) correction within the main outcome families. Simple-effect tests were conducted for significant or theoretically important interactions and were interpreted cautiously when effect sizes were small. Balance checks examined discipline, teaching experience, prior use of AI detection tools, and the post-stimulus acceptance item concerning AI-assisted writing. Because this acceptance item was measured after experimental exposure, it was reported descriptively and was not included as a covariate (Lakens, 2013; Norman, 2010; Richardson, 2011; Simmons et al., 2011). No formal a priori power analysis was conducted; therefore, small interaction effects are interpreted as exploratory and less stable than the large main effects.

Supplementary analyses examined whether the findings depended on data-cleaning decisions, suspicion awareness, correlated quality dimensions, multiple-comparison decisions, or assumption violations. These analyses included re-including recall-check error cases without other exclusion reasons, excluding partially suspicion-aware cases, conducting MANOVA for the three quality indicators, applying FDR-adjusted interpretation to the family of primary ANOVA tests, and using HC3 robust OLS models when homogeneity of variance was not fully met. Factorial ANOVAs were conducted in SPSS 27.0 using Type III sums of squares; supplementary robust models used effect-coded OLS. The study reports p values and partial η^2^ (Berinsky et al., 2014; Brown and Forsythe, 1974; Curran, 2016; DeSimone et al., 2015; Hayes and Cai, 2007; Krosnick, 1991; Lakens, 2013; Long and Ervin, 2000; Meade and Craig, 2012; Norman, 2010; Richardson, 2011; White, 1980).

## 4 Results

This section reports the findings in the order of the analytic strategy: data screening and reliability, manipulation checks, academic-quality evaluations, intervention-related outcomes, and robustness or supplementary analyses. To reduce repetition, detailed auxiliary statistics are retained in Supplementary Appendix, whereas the main text focuses on the results needed to evaluate H1–H5.

### 4.1 Data screening, sample characteristics, and scale reliability

Of the 232 initial responses, 18 were excluded according to the prespecified quality-control rules, yielding 214 valid participants: Group A = 53, Group B = 52, Group C = 55, and Group D = 54. All valid participants passed the attention check and reported relevant grading experience. Post hoc balance checks showed no significant group differences in discipline, χ^2^(9) = 9.368, *p* = 0.404; teaching experience, χ^2^(6) = 8.385, *p* = 0.211; or prior use of AI-detection tools, χ^2^(3) = 0.516, *p* = 0.915. The post-stimulus acceptance item concerning students’ AI-assisted writing differed across groups, *F*(3, 210) = 4.976, *p* = 0.002, but it was measured after exposure to the experimental materials and was therefore reported descriptively rather than used as a covariate. Completion time was used only as an auxiliary cleaning indicator. Descriptive statistics for the main variables are presented in Table 1.

**TABLE 1:** Descriptive statistics of main variables by group (M ± SD).

Variable	A Low AI/no highlighting	B Low AI/red highlighting	C High AI/no highlighting	D High AI/red highlighting
Overall quality rating (1–10)	6.83 (1.05)	6.50 (1.04)	5.02 (0.68)	4.26 (0.65)
Course paper score (0–100)	73.30 (5.21)	71.87 (4.90)	60.64 (4.93)	51.61 (4.35)
Originality mean score	4.39 (0.74)	4.19 (0.77)	2.89 (0.62)	2.12 (0.52)
Language expression mean score	5.73 (0.71)	5.19 (0.73)	4.05 (0.60)	2.89 (0.60)
Logical structure mean score	4.49 (0.67)	4.37 (0.59)	3.53 (0.48)	2.89 (0.58)
Overall multidimensional quality mean	4.87 (0.70)	4.58 (0.68)	3.49 (0.55)	2.63 (0.55)
Behavioral intervention tendency mean	3.40 (0.72)	3.72 (0.79)	4.89 (0.69)	5.66 (0.66)
Perceived AI authorship likelihood	2.23 (0.82)	2.92 (0.99)	5.93 (0.69)	6.35 (0.48)
Perceived AI risk	2.21 (0.79)	2.63 (0.97)	5.84 (0.71)	6.26 (0.62)
AI detection report perception	3.17 (0.44)	3.96 (0.61)	5.30 (0.63)	6.08 (0.58)

All multi-item scales showed acceptable-to-high internal consistency. Cronbach’s αα ranged from 0.817 for behavioral intervention tendency to 0.988 for the overall multidimensional quality scale. For behavioral intervention tendency, the first eigenvalue was 2.247 and explained 74.9% of the variance, with item loadings ranging from 0.722 to 0.939. These results supported averaging the three intervention items into one index. However, the very high α for the overall multidimensional quality scale suggests substantial shared variance among originality, language expression, and logical structure. Therefore, the three quality indicators are interpreted primarily as closely related analytic indicators of a broader evaluative judgment rather than as fully discriminant latent constructs. Dimension-specific findings, especially those related to H4, are consequently treated as descriptive rather than as evidence of clearly separable quality dimensions.

### 4.2 Manipulation checks

The manipulations were effective. For perceived AI authorship likelihood, the main effect of algorithmic warning was significant, *F*(1, 210) = 1160.260, *p* < 0.001, ηp^2^ = 0.847, indicating that participants in the high-rate condition judged the paper as more likely to be AI-authored or AI-assisted. The visual-risk/report-presentation cue also had a significant main effect, *F*(1, 210) = 28.696, *p* < 0.001, ηp^2^ = 0.120. The interaction was not significant, *F*(1, 210) = 1.690, *p* = 0.195, ηp^2^ = 0.008. Group means increased from Group A (*M* = 2.23) to Group B (*M* = 2.92), Group C (*M* = 5.93), and Group D (*M* = 6.35).

Perceived AI risk showed the same pattern. Algorithmic warning had a significant main effect, *F*(1, 210) = 1147.965, *p* < 0.001, ηp^2^ = 0.845, and the visual-risk/report-presentation cue also had a significant main effect, *F*(1, 210) = 15.763, *p* < 0.001, ηp^2^ = 0.070. The interaction was not significant, *F*(1, 210) = 0.000, *p* = 0.984, ηp^2^ = 0.000. Group means were 2.21, 2.63, 5.84, and 6.26 for Groups A–D. Thus, the AI-rate manipulation created a strong perceived-risk contrast, and red highlighting with review-oriented wording further increased perceived AI relevance even when the numerical warning was held constant.

### 4.3 Effects on academic quality evaluation

For the 1–10 overall quality rating, algorithmic warning significantly lowered evaluations, *F*(1, 210) = 288.523, *p* < 0.001, ηp^2^ = 0.579. The visual-risk/report-presentation cue also had a significant main effect, *F*(1, 210) = 20.837, *p* < 0.001, ηp^2^ = 0.090. The interaction was not statistically significant at α = 0.05, *F*(1, 210) = 3.229, *p* = 0.074, ηp^2^ = 0.015. Group means were 6.83, 6.50, 5.02, and 4.26 for Groups A–D, showing the same descriptive pattern as the other quality outcomes but with weaker evidence for interaction.

The 0–100 course paper score was more sensitive to the combined cues. Algorithmic warning, visual cue, and their interaction were all significant: *F*(1, 210) = 615.055, *p* < 0.001, ηp^2^ = 0.745; *F*(1, 210) = 62.117, *p* < 0.001, ηp^2^ = 0.228; and *F*(1, 210) = 32.684, *p* < 0.001, ηp^2^ = 0.135, respectively. The red-highlighting/report-presentation cue did not significantly change scores under the low-AI condition, but it significantly reduced scores under the high-AI condition, suggesting selective amplification when visual evidence cues were embedded in a strong algorithmic-warning context.

The multidimensional quality outcomes showed a convergent pattern. For originality, algorithmic warning, visual cue, and their interaction were significant, *F*(1, 210) = 385.812, *p* < 0.001, ηp^2^ = 0.648; *F*(1, 210) = 28.492, *p* < 0.001, ηp^2^ = 0.119; and *F*(1, 210) = 10.021, *p* = 0.002, ηp^2^ = 0.046. For language expression, the corresponding effects were also significant, *F*(1, 210) = 483.059, *p* < 0.001, ηp^2^ = 0.697; *F*(1, 210) = 88.555, *p* < 0.001, ηp^2^ = 0.297; and *F*(1, 210) = 11.580, *p* = 0.001, ηp^2^ = 0.052. For logical structure, the effects were significant as well, *F*(1, 210) = 235.551, *p* < 0.001, ηp^2^ = 0.529; *F*(1, 210) = 22.567, *p* < 0.001, ηp^2^ = 0.097; and *F*(1, 210) = 10.628, *p* = 0.001, ηp^2^ = 0.048.

**FIGURE 1:**
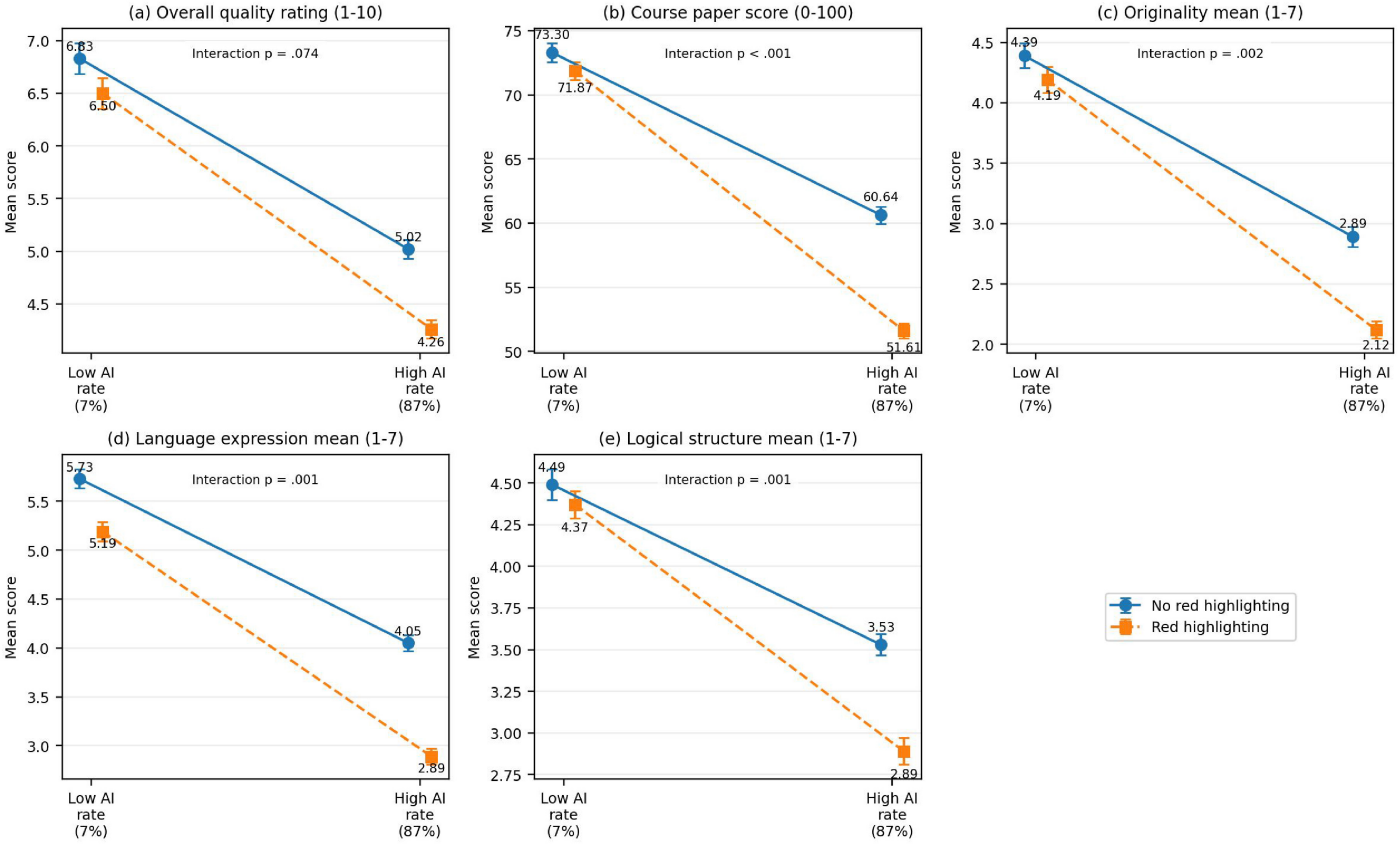
Effects of AI detection rate and red-highlighting/report-presentation package on academic quality evaluations: **(a)** overall quality rating, **(b)** course paper score, **(c)** originality, **(d)** language expression, and **(e)** logical structure. Points represent group means, and error bars represent SE.

Simple-effect patterns clarified the interaction. Under the low-AI condition, red highlighting significantly lowered language-expression evaluations but did not significantly affect originality or logical structure. Under the high-AI condition, red highlighting significantly lowered all three multidimensional quality evaluations. The visual-cue main effect was largest for language expression (partial η^2^ = 0.297), compared with originality (0.119) and logical structure (0.097). This ordering is consistent with H4’s expectation that visual risk cues are most closely tied to surface language evaluation. However, because discriminant validity among the three quality indicators was not established and the study did not formally test differences among effect sizes across dependent variables, this pattern is interpreted descriptively rather than as a definitive dimension-specific mechanism. Figure 1 displays the academic-quality means, and Table 2 consolidates the corresponding ANOVA results.

**TABLE 2:** 2 × 2 ANOVA results for academic quality evaluation variables.

Dependent variable	Effect	df	*F*	*p*	Partial η ^2^
Overall quality rating (1–10)	Main effect of algorithmic warning	1, 210	288.523	<0.001	0.579
Overall quality rating (1–10)	Main effect of visual highlighting	1, 210	20.837	<0.001	0.090
Overall quality rating (1–10)	Interaction effect	1, 210	3.229	0.074	0.015
Course paper score (0–100)	Main effect of algorithmic warning	1, 210	615.055	<0.001	0.745
Course paper score (0–100)	Main effect of visual highlighting	1, 210	62.117	<0.001	0.228
Course paper score (0–100)	Interaction effect	1, 210	32.684	<0.001	0.135
Originality mean score	Main effect of algorithmic warning	1, 210	385.812	<0.001	0.648
Originality mean score	Main effect of visual highlighting	1, 210	28.492	<0.001	0.119
Originality mean score	Interaction effect	1, 210	10.021	0.002	0.046
Language expression mean score	Main effect of algorithmic warning	1, 210	483.059	<0.001	0.697
Language expression mean score	Main effect of visual highlighting	1, 210	88.555	<0.001	0.297
Language expression mean score	Interaction effect	1, 210	11.580	0.001	0.052
Logical structure mean score	Main effect of algorithmic warning	1, 210	235.551	<0.001	0.529
Logical structure mean score	Main effect of visual highlighting	1, 210	22.567	<0.001	0.097
Logical structure mean score	Interaction effect	1, 210	10.628	0.001	0.048
Overall multidimensional quality mean	Main effect of algorithmic warning	1, 210	383.876	<0.001	0.646
Overall multidimensional quality mean	Main effect of visual highlighting	1, 210	45.222	<0.001	0.177
Overall multidimensional quality mean	Interaction effect	1, 210	11.249	0.001	0.051

*p*-values are reported as exact or thresholded values for transparency. Because multiple related outcomes were tested, the interpretation of the primary ANOVA findings also considered Benjamini–Hochberg FDR correction within the main outcome families. Marginal or non-significant results, such as the *p* = 0.074 interaction for the 1–10 overall quality rating, were not interpreted as evidence of an interaction.

### 4.4 Effects on intervention tendency and report perception

For behavioral intervention tendency, both algorithmic warning and visual cue had significant main effects, *F*(1, 210) = 307.768, *p* < 0.001, ηp^2^ = 0.594, and *F*(1, 210) = 31.024, *p* < 0.001, ηp^2^ = 0.129. Their interaction was also significant, *F*(1, 210) = 5.225, *p* = 0.023, ηp^2^ = 0.024. Group means were 3.40, 3.72, 4.89, and 5.66 for Groups A–D. Simple effects showed that red highlighting slightly increased intervention tendency under low AI (A vs. B: *t* = -2.178, *p* = 0.032, *d* = 0.425) and more strongly under high AI (C vs. D: *t* = -5.949, *p* < 0.001, *d* = 1.140). These results support H5, but the interaction should be interpreted cautiously because its effect size was small and the measure captured stated intention rather than observed teaching behavior.

**FIGURE 2:**
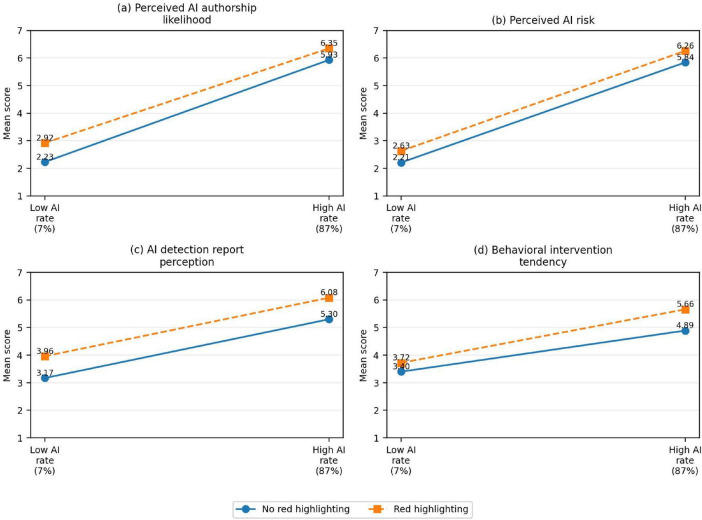
Effects of AI detection rate and red-highlighting/report-presentation package on manipulation checks and behavioral responses: **(a)** perceived AI authorship likelihood, **(b)** perceived AI risk, **(c)** AI detection report perception, and **(d)** behavioral intervention tendency. Points represent group means, and error bars represent SE.

For AI detection report perception, algorithmic warning and visual cue had significant main effects, *F*(1, 210) = 748.158, *p* < 0.001, ηp^2^ = 0.781, and *F*(1, 210) = 102.147, *p* < 0.001, ηp^2^ = 0.327. The interaction was not significant, *F*(1, 210) = 0.001, *p* = 0.977, ηp^2^ = 0.000. Group means were 3.17, 3.96, 5.30, and 6.08 for Groups A–D. Thus, red highlighting increased perceived report influence under both low- and high-warning conditions, but it operated as a stable main effect rather than as a warning-dependent interaction for this outcome. Figure 2 summarizes the non-quality outcomes, and Table 3 reports the simple effects of the red-highlighting/report-presentation cue.

**TABLE 3:** Simple effects of the red-highlighting/report-presentation condition for key dependent variables.

Dependent variable	Comparison	*t*	*P*	Cohen’s *d*
Overall quality rating (1–10)	A vs. B	1.619	0.109	−0.316
Overall quality rating (1–10)	C vs. D	5.955	<0.001	−1.141
Course paper score (0–100)	A vs. B	1.456	0.148	−0.284
Course paper score (0–100)	C vs. D	10.127	<0.001	−1.940
Originality mean score	A vs. B	1.346	0.181	−0.263
Originality mean score	C vs. D	7.090	<0.001	−1.358
Language expression mean score	A vs. B	3.867	<0.001	−0.755
Language expression mean score	C vs. D	10.086	<0.001	−1.932
Logical structure mean score	A vs. B	0.968	0.335	−0.189
Logical structure mean score	C vs. D	6.231	<0.001	−1.194
Behavioral intervention tendency mean	A vs. B	−2.178	0.032	0.425
Behavioral intervention tendency mean	C vs. D	−5.949	<0.001	1.140
AI detection report perception mean	A vs. B	−7.594	<0.001	1.482
AI detection report perception mean	C vs. D	−6.794	<0.001	1.302

Cohen’s *d* was calculated as Group 2 minus Group 1; negative values indicate lower scores in the red-highlighting/report-presentation condition, and positive values indicate higher scores. These simple-effect estimates are reported to describe the interaction pattern and should be interpreted cautiously because the 7% vs. 87% contrast was intentionally strong, several effects are small, and confidence intervals for these point estimates should be added in future supplementary reporting where raw-data reanalysis is available.

### 4.5 Robustness and supplementary analyses

The main conclusions were stable across robustness checks. For outcomes where Levene’s tests indicated incomplete homogeneity, HC3 robust OLS models reproduced the substantive conclusions of the factorial ANOVAs. Re-including recall-check error cases without other exclusion reasons increased the sample to *N* = 218 and did not change the main effects or the significance pattern of the core interactions. Excluding the four partially suspicion-aware cases reduced the sample to *N* = 210 and likewise preserved the main conclusions. Applying Benjamini–Hochberg FDR correction to the primary ANOVA outcome family did not change the central interpretation: strong main effects remained robust, the *p* = 0.074 interaction for the 1–10 rating remained non-significant, and the reported interaction evidence was retained mainly for percentage-based scoring, multidimensional quality indicators, and intervention tendency. These checks suggest that the findings were not driven by the main data-cleaning decisions, the small number of partially suspicion-aware responses, or uncorrected interpretation alone.

Supplementary analyses were consistent with the primary results but should be interpreted cautiously. Exploratory mediation models suggested that perceived AI authorship likelihood may be one plausible pathway linking algorithmic warning to lower quality evaluations and stronger intervention tendency. However, perceived AI authorship and quality judgments were measured at the same post-stimulus time point, and the mediator was highly condition-linked; therefore, the direction of the proposed pathway cannot be established. The mediation evidence is therefore treated as suggestive mechanism-oriented evidence rather than as a strict causal test. Future studies should temporally separate mediator and outcome measurement, for example by measuring perceived AI authorship immediately after report exposure and reassessing quality judgments 1 week later. A 2 × 2 MANOVA using originality, language expression, and logical structure as joint dependent variables showed significant multivariate effects of algorithmic warning, visual highlighting, and their interaction, supporting the univariate multidimensional-quality findings while not establishing discriminant validity among the three indicators. Open-ended response coding also converged descriptively with the quantitative pattern, but these open-ended findings are treated as supplementary because the category structure and reliability coefficients should be reported more fully in future or supplementary documentation.

Overall, the results support H1, H2, and H5, provide selective support for H3, and offer descriptive support for H4. A high AI detection rate consistently lowered quality evaluations and increased intervention tendency. The red-highlighting/report-presentation cue also influenced judgment, especially language evaluation and report perception, and it amplified the high-warning effect for percentage-based scoring, closely related multidimensional quality indicators, and intervention tendency. The pattern was not uniform across all outcomes: the interactions for the 1–10 overall rating and AI detection report perception were not statistically significant.

## 5 Discussion

### 5.1 Main findings

Using a 2 × 2 between-subjects experiment, this study examined how algorithmic warnings and visual risk cues in AI detection reports influence teachers’ evaluations of student writing in a controlled, single-stimulus setting. A high AI detection rate significantly increased perceived AI authorship likelihood and risk, and significantly lowered evaluations of the same paper’s overall quality, originality, language expression, and logical structure. The red-highlighting/report-presentation package also affected judgments, particularly language expression and report perception. These findings are consistent with Figures 1, 2 and Tables 1–3, but they should be interpreted as context-specific evidence from one Chinese social-science paper rather than as definitive evidence for all detection-report settings.

Algorithmic warning and visual risk cue interacted for some evaluation indicators. Under low AI, the red-highlighting condition had weak or non-significant effects on overall quality, originality, and logical structure. Under high AI, it further reduced percentage-based scores, originality, language expression, logical structure, and overall multidimensional quality, and it more strongly increased behavioral intervention tendency. Thus, the visual-risk-cue condition became more influential when embedded in a high-warning context, where it could serve as visual evidence supporting the system judgment and prompting stronger intervention-oriented responses.

The interaction pattern was not uniform across all dependent variables. The interaction for overall quality rating and report perception was non-significant. A more precise interpretation is therefore that the red-highlighting/report-presentation condition produced selective amplification under high-AI conditions, especially for percentage-based scoring, multidimensional quality, and intervention-oriented responses, rather than a uniform interaction across all outcomes.

The two overall evaluation indicators also differed in sensitivity. The warning × highlighting interaction was significant for the 0–100 course paper score but not statistically significant for the 1–10 overall rating, likely because the 1–10 scale compressed variation while the 0–100 score allowed finer grading distinctions. Both indicators nevertheless showed the same descriptive pattern: the red-highlighting/report-presentation condition had little effect under low AI but a clear negative effect under high AI.

The large partial η^2^ values for algorithmic warning should be interpreted cautiously. They partly reflect the strong contrast between 7 and 87% detection rates and the limited contextual information available in the experiment. In real teaching, teachers may see intermediate rates and have richer information about students and writing processes, so actual bias may be smaller.

### 5.2 Automation bias: from reference tool to judgment anchor

The findings can be explained by automation bias. When teachers encounter an AI detection report, they may treat the system output as objective. The label “AI content: 87%” can anchor judgments of both source and quality. Because the paper content was identical across groups, lower scores in high-AI conditions show that evaluations were shaped by external algorithmic cues, not by text alone.

This does not mean that teachers abandon independent judgment. Rather, algorithmic cues shift the starting frame from “evaluating paper quality” to “checking whether the system cue is valid.” This framing shift is a key mechanism through which AI detection reports affect educational evaluation.

### 5.3 Visual risk cues and confirmatory interpretation

The red-highlighting condition effect shows that report interface design is not neutral. The highlighted sentences were ordinary formulaic expressions without clear factual errors or logical breaks, yet system marking made them easier to read as signs of AI generation.

Language expression results especially support this interpretation. Even under low AI, the red-highlighting condition lowered language-expression scores. Its partial η^2^ for language expression (0.297) exceeded those for originality (0.119) and logical structure (0.097), suggesting that highlighting first affected perceptions of surface-level language. Under high AI, this suspicion spilled over to originality, logical structure, and overall quality.

Thus, the red-highlighting condition may do more than locate problems; it can induce confirmatory search. After accepting the possibility of AI generation, teachers may selectively attend to formulaic wording and reinterpret ordinary expressions as suspicious.

### 5.4 From evaluation bias to behavioral consequences

AI detection reports also affected self-reported behavioral intervention tendency. High AI rate and the red-highlighting condition increased teachers’ stated likelihood of requiring revision, reminding students about writing independence, and requesting process explanations. Group D showed the highest intervention tendency, and the significant interaction indicates that red highlighting produced a stronger increase in intervention-oriented responses when paired with a high AI detection rate.

This indicates that detection reports may influence not only quality scores but also anticipated teaching responses. Because the study measured intentions rather than observed behavior, field research is needed to test whether similar patterns occur in actual grading, feedback, appeals, and teacher-student interactions.

### 5.5 Theoretical contributions

This study makes three cautious theoretical contributions. First, it extends automation-bias reasoning to AI text detection and educational evaluation. Whereas prior work often focuses on medicine, aviation, driving, and industrial decision-making, this controlled experiment suggests that automated outputs may also shape everyday educational judgment when teachers evaluate student writing under uncertainty.

Second, the study separates algorithmic warnings from visual risk cues. Existing discussions often treat AI detection reports as a single stimulus; the 2 × 2 design suggests that detection-rate and red-highlighting/report-presentation conditions can have independent effects and can interact for percentage-based scoring, multidimensional quality indicators, and behavioral intervention tendency. The influence of detection reports therefore appears to arise from both algorithmic conclusions and interface presentation, although replication with other report formats is needed.

Third, the study identifies a descriptive dimension-level pattern. The red-highlighting condition most strongly affected language expression, while its effects on overall quality, originality, and logical structure depended more on the high-AI context. Because discriminant validity among the three quality indicators was not established, this pattern should be understood as variation among closely related rating indicators rather than as proof that three independent quality constructs were differentially affected.

The study is also consistent with a possible sequence linking automation bias and confirmatory interpretation, but it does not prove this temporal chain. Automation bias may appear when teachers treat high AI-rate numbers and risk labels as authoritative, increasing perceived AI authorship and weakening independently formed quality judgments. Confirmation-oriented reading may then follow when the red-highlighting condition supplies local evidence targets and encourages selective attention to formulaic sentences. Because the mediator and outcomes were measured simultaneously, this proposed chain of “algorithmic warning forming suspicion—visual cues triggering confirmatory search—evaluation declining and intervention tendency increasing” should be treated as a theoretical interpretation to be tested with temporally separated or process-tracing designs.

### 5.6 Practical implications

This study has practical implications for universities, but these implications should be applied cautiously because the evidence comes from a controlled experiment using one stimulus paper. First, AI detection reports should not be treated as direct evidence of writing independence. They should be auxiliary cues, not substitutes for teachers’ comprehensive assessment of content, argumentation, and writing process.

Second, universities could consider implementing a two-stage procedure in which teachers complete their quality evaluation before reviewing the detection report, and then use the report only as Supplementary review material. This can reduce anchoring by algorithmic warnings.

Third, report interfaces should avoid excessive threat cues. Strong red colors, warning icons, and high-risk labels, especially when paired with prompt wording that frames passages as review-worthy, may amplify evaluation bias. Developers and administrators can use more neutral wording, such as “manual review recommended” rather than “high risk” or “suspected AI-generated.”

Fourth, teacher training should address automation bias and confirmation bias so that teachers remain aware of system influence and maintain independent judgment when using AI detection tools. In addition, recent classroom evidence on LLM-assisted grading and appeal resolution highlights the need for transparent rubrics, human oversight, and appeal mechanisms when automated evaluation tools are used in educational assessment (Aytutuldu et al., 2025).

### 5.7 Limitations and future directions

This study has several limitations. First, it used a fictitious detection report, an online experimental setting, and a single medium-quality Chinese social-science paper. Although this design improved experimental control, it limits ecological and external validity. Moreover, the medium-quality level of the stimulus paper was not independently validated through a pilot study or external rater calibration. The effects of detection reports might differ for very high-quality or very poor papers, where teachers’ prior quality expectations could moderate the influence of algorithmic warnings and visual risk cues. Future research should replicate the findings with real detection interfaces, real grading procedures, independently validated stimulus materials, and papers varying in discipline, language, topic, and quality level.

Second, several design and sample features require caution. Some variables approached scale endpoints, which may have reduced sensitivity to additional cue effects and interactions. Although discipline, teaching experience, and prior AI-detection-tool use were approximately balanced across groups in post hoc checks, the post-stimulus acceptance item concerning students’ AI-assisted writing differed across groups. Because that item was measured after stimulus exposure, it was not used as a covariate and is reported descriptively only. Future studies could measure prior AI attitudes before stimulus exposure and use stratified or blocked randomization to improve balance on key background variables.

Third, the visual-risk-cue manipulation should be interpreted as a report-presentation package rather than as a pure color-only manipulation, because the red-highlighting conditions were accompanied by condition-consistent prompt wording referring to highlighted or review-worthy passages. Future research should orthogonally separate color highlighting from accompanying textual prompts, for example by crossing color, sentence-level marking, and prompt wording independently.

Fourth, measurement and mechanism evidence remain limited. High Cronbach’s α values may partly reflect similar item wording and a shared global evaluation of the paper rather than broad construct coverage or clear discriminant validity. Therefore, dimension-specific interpretations, especially H4, should be treated as descriptive. Future research should report intercorrelation matrices and confirmatory factor analyses for quality dimensions, develop broader measures with more diverse item content, avoid artificially inflated reliability from item homogeneity, and distinguish subdimensions within each construct–for example, separating novelty of ideas from uniqueness of argumentation within originality. The mediation analysis was exploratory and self-report-based. More importantly, perceived AI authorship and quality judgments were measured simultaneously, so the direction of the proposed pathway cannot be established. Future studies should separate mediator and outcome measurement over time and combine preregistered coding manuals, transparent open-ended coding categories, inter-coder reliability coefficients, process tracing, eye tracking, interviews, or other methods. A small number of extreme or internally inconsistent response patterns (for example, high overall quality ratings combined with very high AI-authorship-likelihood judgments in high-warning conditions) were excluded only when they met the prespecified abnormal or contradictory response-pattern criteria; future research could define such careless-response indicators more stringently a priori.

Finally, this study measured immediate self-reported evaluations and intended interventions rather than actual teaching behavior. Field experiments with larger samples and multiple papers are needed to examine whether AI detection reports affect real scores, feedback, appeals, and teacher-student interactions, and to test moderated mediation involving the warning × highlighting interaction. In addition, no formal a priori power analysis was conducted before data collection; therefore, small interaction effects such as ηp^2^ values near 0.015–0.024 should be interpreted as exploratory and should be tested in future adequately powered replications.

## 6 Conclusion

This study examined how algorithmic warning strength and visual risk cues in AI detection reports influence teachers’ evaluations of student writing in a controlled single-stimulus experiment. Using a 2 × 2 between-subjects design with the student paper held constant, it found that a high AI detection rate lowered evaluations of the same paper’s overall quality, originality, language expression, and logical structure, and increased self-reported intervention-oriented responses. More importantly, the red-highlighting/report-presentation condition selectively amplified the effect of high AI detection rates for several outcomes. Significant warning × highlighting interactions emerged for percentage-based scoring, originality, language expression, logical structure, overall multidimensional quality, and behavioral intervention tendency. However, this amplification was not uniform across all outcomes: the interactions for the 1–10 overall rating and AI detection report perception were not statistically significant.

In this controlled context, these findings suggest that AI detection reports may function not merely as neutral technical outputs, but as socio-technical judgment environments. The detection percentage provides a global algorithmic warning, whereas the red-highlighting/report-presentation condition provides local visual evidence targets. When these cues appear together, teachers may be more likely to treat ordinary formulaic expressions as evidence of AI involvement, leading to lower evaluations of several writing-quality indicators and stronger self-reported intentions to intervene. The exploratory mediation analysis further suggests that perceived AI authorship likelihood may be one plausible psychological pathway through which algorithmic warnings shape evaluative judgments and intervention-oriented responses. However, this pathway should be interpreted as supplementary mechanism-oriented evidence rather than as strict causal mediation because mediator and outcome measures were collected at the same time point.

The core implication is that AI text detection reports can shape teachers’ self-reported evaluative judgments under controlled experimental conditions, especially when strong numerical warnings are paired with visually salient risk cues. Universities should therefore avoid treating detection reports as decisive evidence and should reduce avoidable bias through independent evaluation before report review, less threatening report design, transparent rubrics, human oversight, appeal mechanisms, and teacher training on automation bias and confirmation bias. Because this study used a strong manipulation, a single medium-quality Chinese paper, and intention-based measures, the findings should not be generalized directly to all real grading contexts. They nonetheless indicate that the responsible use of AI detection tools requires attention not only to detector accuracy, but also to how report interfaces guide human judgment.

## Data Availability

The anonymized data supporting this study are available from the corresponding author upon reasonable request.
