# Supplementary material for: Automation bias in teachers’ evaluation of student writing: effects of algorithmic warnings and visual risk cues in AI detection reports
Source: Front Psychol. 2026 Jul 7;17:1889402. doi: 10.3389/fpsyg.2026.1889402 (PMC13387046; doi:10.3389/fpsyg.2026.1889402)
Supplement: Supplementary file 1 [file Supplementary_file_1.docx]

**Appendices**

The appendices provide experimental materials, questionnaire scales, variable coding, and supplementary notes. Depending on journal requirements, some may be moved to online supplementary materials.

**Appendix A. English Translation of the Student Course Paper Used in the Experiment**

Title: The Effects and Challenges of Online Education

With the rapid development of information technology, online education has gradually become an important component of higher education. In recent years, more and more universities have begun to incorporate live-streamed courses, recorded resources, and online discussion platforms into everyday teaching. Compared with traditional classrooms, online education breaks the constraints of fixed classrooms and fixed times, allowing students to arrange course content according to their own learning pace. While this change improves learning convenience, it also changes students' learning methods and patterns of teacher-student interaction. This paper analyzes this issue from three aspects: learning effects, interaction modes, and technical conditions.

First, online education has relatively clear advantages in learning convenience. Students can repeatedly watch course recordings to review knowledge points they did not understand, and they can also use fragmented time to complete some learning tasks. For university students who have long commutes, participate in internships, or need to balance other responsibilities, online courses indeed offer considerable flexibility. However, the effectiveness of online education does not depend entirely on the platform itself. Some students lack self-management ability and are easily distracted without classroom supervision; some may even play the course while doing other tasks. Therefore, whether online education achieves good results also depends jointly on students' self-discipline, teachers' instructional design, and course evaluation methods.

Second, online education changes interaction between teachers and students and among students. In traditional classrooms, teachers can adjust the pace of teaching in time based on students' facial expressions, eye contact, and classroom reactions, whereas such feedback is often less direct in online classrooms. Although comments, discussion boards, and online Q&A can provide some channels for interaction, these forms are sometimes fragmented and may not lead to in-depth discussion. Some students are more willing to speak in online environments because they face less pressure, while others remain silent for long periods and passively receive course content. Thus, online education is not simply the transfer of classrooms to the internet; it requires redesigned interaction modes to avoid turning teaching into one-way information delivery.

Third, technical conditions are also important factors affecting the quality of online education. Stable internet connections, clear video resources, and easy-to-use platforms all affect students' learning experience. If the platform frequently lags or course materials are distributed across multiple systems, students may develop fatigue and confusion. At the same time, students' device conditions and network environments are not identical, which may create differences in learning opportunities. For universities, promoting online education should not focus only on platform construction; it must also consider supporting measures such as teacher training, course management, and student support services.

In conclusion, online education brings convenience but also faces many practical challenges. It can improve access to learning resources and promote changes in teaching forms to some extent, but it may also lead to insufficient self-discipline, reduced interaction quality, and uneven technical conditions. When developing online education in the future, universities should maintain a balance between technological application and pedagogical principles, neither simply denying the value of online education nor over-relying on technological platforms themselves. Therefore, this issue deserves continued attention and further research from the education community.

Target sentences in the red-highlighting conditions (the text content was identical across all four groups; in Groups B and D, the following four sentences were displayed in red, while in Groups A and C they remained ordinary black text. For formatting consistency, this appendix lists only the sentence text):

- With the rapid development of information technology, online education has gradually become an important component of higher education.
- This paper analyzes this issue from three aspects: learning effects, interaction modes, and technical conditions.
- In conclusion, online education brings convenience but also faces many practical challenges.
- Therefore, this issue deserves continued attention and further research from the education community.

Note. The original experimental material was presented to participants in Chinese. This appendix provides an English translation for review purposes. If required by the journal, the original Chinese stimulus materials can be provided as an additional online supplementary file or made available upon request.

**Appendix B. Four Versions of the AI Detection Report Materials**

Appendix Table B1. AI Detection Report Conditions for the Four Groups

| **Condition** | **AI content** | **Visual highlighting** | **Report prompt** |
| --- | --- | --- | --- |
| Group A: low AI rate/no highlighting | 7% | None | Low risk: The text shows predominantly human-authored writing characteristics. No immediate concern is indicated. |
| Group B: low AI rate/red highlighting | 7% | Four sentences highlighted in red | Low risk: The text shows predominantly human-authored writing characteristics. Some passages may benefit from manual review due to formulaic phrasing. |
| Group C: high AI rate/no highlighting | 87% | None | High risk: The text shows a high likelihood of AI-assisted writing. Manual review is recommended. |
| Group D: high AI rate/red highlighting | 87% | Four sentences highlighted in red | High risk: The text shows a high likelihood of AI-assisted writing. Flagged passages are highlighted below for manual review. |

**Appendix C. Student Paper Evaluation Questionnaire Scale (Formal Version Shared by All Four Groups)**

Instructions: Please respond based on your reading of the student course paper and the system report. Unless otherwise specified, 1 = strongly disagree and 7 = strongly agree.

C1. Overall Academic Quality Evaluation

- Q1. Please rate the overall academic quality of this paper. (1 = very poor, 10 = excellent)
- Q2. If this were a course paper, what score would you assign? (0-100 points)

C2. Multidimensional Academic Quality Evaluation

- Q3. This paper presents relatively independent viewpoints.
- Q4. This paper shows the author's independent thinking about the issue.
- Q5. The arguments in this paper have some degree of novelty.
- Q6. The language expression in this paper is generally fluent.
- Q7. The wording in this paper basically conforms to the writing conventions of course papers.
- Q8. The language expression in this paper has natural writing characteristics.
- Q9. The argumentative structure of this paper is relatively clear.
- Q10. The sections of this paper are basically logically coherent.
- Q11. The main arguments of this paper are basically supported.

C3. Behavioral Tendency

- Q12. I would require this student to further revise the paper. (1 = definitely would not, 7 = definitely would)
- Q13. I would remind this student to pay attention to writing independence or AI-use norms. (1 = definitely would not, 7 = definitely would)
- Q14. I would require this student to provide additional explanation of the writing process or sources. (1 = definitely would not, 7 = definitely would)

C4. Manipulation Checks

- Q15. How likely do you think this paper is to have been generated or assisted by AI? (1 = completely impossible, 7 = almost certain)
- Q16. How high do you think the AI risk level shown by the detection report is? (1 = very low, 7 = very high)
- Q17. Please recall: approximately what AI content value was shown in the detection report? A. 7%; B. 37%; C. 87%; D. I do not remember

C5. AI Detection Report Perception

- Q18. I think this AI detection report provides useful reference information.
- Q19. This detection report influenced my judgment of the source of the paper.
- Q20. This detection report made me pay more attention to possible formulaic expressions in the text.
- Q21. When evaluating this paper, I referred to the risk cues in the detection report.
- Q22. Even if the detection report indicates risk, I would still mainly rely on the paper content itself in making my judgment. (reverse scored)

C6. Open-Ended Item, Suspicion Probe, and Attention Check

- Q23. If this were a student paper you were supervising or grading, what specific revision suggestions would you give?
- Q24. What do you think the true purpose of this study is?
- Q25. To confirm that you are responding carefully, please select "somewhat agree" for this item. (Correct option: 6 = somewhat agree)

C7. Demographics and Background Variables

- Q26. Your disciplinary field: humanities / social sciences / science and engineering / medicine / other
- Q27. Your teaching experience: 1-3 years / 4-10 years / 11-20 years / more than 20 years
- Q28. Have you ever used an AI text detection or AI plagiarism-checking tool? Yes / No
- Q29. Your acceptance of students' use of AI-assisted writing in course papers is: 1 = completely unacceptable, 7 = completely acceptable
- Q30. Do you have experience grading course papers, theses, or student assignments? Yes / No

**Appendix D. Variable Coding and Scoring Instructions**

Appendix Table D1. Variable Coding and Scoring Instructions

| **Variable/item** | **Coding or scoring method** |
| --- | --- |
| condition | A = low AI rate 7% + no highlighting; B = low AI rate 7% + red highlighting; C = high AI rate 87% + no highlighting; D = high AI rate 87% + red highlighting |
| warning | A/B = 0; C/D = 1 |
| cue | A/C = 0; B/D = 1 |
| Originality mean score | Mean of Q3, Q4, and Q5 |
| Language expression mean score | Mean of Q6, Q7, and Q8 |
| Logical structure mean score | Mean of Q9, Q10, and Q11 |
| Overall multidimensional quality mean | Mean of nine items from Q3 to Q11 |
| Behavioral intervention tendency mean | Mean of Q12, Q13, and Q14 |
| AI detection report perception mean | Mean of Q18, Q19, Q20, Q21, and reverse-scored Q22; reversed Q22 = 8 - Q22 |
| Manipulation checks | Q15 perceived AI authorship likelihood; Q16 perceived AI risk; Q17 AI rate recall item |
| Main analysis method | Effect-coded Type III 2 × 2 ANOVA/OLS: warning, cue, warning × cue; report F, p, and partial η² |
| Exclusion criteria | Q25 attention-check failure, abnormal completion time, invariant responses on Q3-Q22, clear Q17 AI-rate recall-check errors indicating misidentification of the manipulated rate, contradiction between Q15/Q16 or Q15 and experimental condition, Q24 suspicion probe; Q23 open-ended response quality as an auxiliary criterion. Minor uncertainty responses to Q17 were retained when Q15/Q16 were consistent with the assigned condition. |

**Appendix E. Supplementary Statistical Results**

This appendix provides full ANOVA results for manipulation checks, behavioral intervention tendency, and AI detection report perception. These results are summarized in the main text and reported here in table form for completeness. See Appendix Tables E1 and E2.

Appendix Table E1. Manipulation Check Results

| **Dependent variable** | **Effect** | **df** | **F** | **p** | **partial η²** |
| --- | --- | --- | --- | --- | --- |
| Perceived AI authorship likelihood | Main effect of algorithmic warning | 1, 210 | 1160.260 | <.001 | 0.847 |
| Perceived AI authorship likelihood | Main effect of visual highlighting | 1, 210 | 28.696 | <.001 | 0.120 |
| Perceived AI authorship likelihood | Interaction effect | 1, 210 | 1.690 | .195 | 0.008 |
| Perceived AI risk | Main effect of algorithmic warning | 1, 210 | 1147.965 | <.001 | 0.845 |
| Perceived AI risk | Main effect of visual highlighting | 1, 210 | 15.763 | <.001 | 0.070 |
| Perceived AI risk | Interaction effect | 1, 210 | 0.000 | .984 | 0.000 |

Appendix Table E2. Behavioral Intervention Tendency and Report Perception Results

| **Dependent variable** | **Effect** | **df** | **F** | **p** | **partial η²** |
| --- | --- | --- | --- | --- | --- |
| Behavioral intervention tendency mean | Main effect of algorithmic warning | 1, 210 | 307.768 | <.001 | 0.594 |
| Behavioral intervention tendency mean | Main effect of visual highlighting | 1, 210 | 31.024 | <.001 | 0.129 |
| Behavioral intervention tendency mean | Interaction effect | 1, 210 | 5.225 | .023 | 0.024 |
| AI detection report perception mean | Main effect of algorithmic warning | 1, 210 | 748.158 | <.001 | 0.781 |
| AI detection report perception mean | Main effect of visual highlighting | 1, 210 | 102.147 | <.001 | 0.327 |
| AI detection report perception mean | Interaction effect | 1, 210 | 0.001 | .977 | 0.000 |

Note. Appendix Table E2 reports partial eta-squared for all behavioral-intervention and report-perception effects, including the behavioral-intervention interaction.

**Appendix F. Supplementary Analysis Notes**

Appendix Table F1. Sensitivity Analysis After Including Recall-Check Error Cases

| **Dependent variable** | **Algorithmic warning F(p)** | **Visual highlighting F(p)** | **Interaction F(p)** |
| --- | --- | --- | --- |
| Overall quality rating (1-10) | 294.782 (<.001) | 21.437 (<.001) | 3.494 (.063) |
| Course paper score (0-100) | 627.717 (<.001) | 64.083 (<.001) | 34.531 (<.001) |
| Overall multidimensional quality mean | 396.317 (<.001) | 45.412 (<.001) | 11.892 (<.001) |
| Behavioral intervention tendency mean | 321.158 (<.001) | 32.688 (<.001) | 5.647 (.018) |

Note: This analysis re-included cases explicitly marked as clear AI-rate recall-check errors and without any other exclusion reason, while all other excluded cases remained excluded. In the analysis workbook, clear recall-check errors were identified by a mismatch between Q17 and the assigned report value and recorded in the exclusion column as recall-error cases. After re-inclusion, group sizes were A = 54, B = 53, C = 56, and D = 55, with total N = 218. The results were used to examine whether the data-cleaning decision affected the core conclusions. Minor uncertainty responses, including selection of the 'I do not remember' option, were not treated as recall-check errors when other manipulation-check responses were consistent with the assigned condition.

Appendix Table F2. Mediation Analysis of Perceived AI Authorship Likelihood

| **Dependent variable** | **a path** | **b path** | **Indirect effect** | **95% CI** | **Interpretation** |
| --- | --- | --- | --- | --- | --- |
| Overall quality rating (1-10) | 3.701 | -0.982 | -3.635 | [-4.047, -3.229] | Significant negative mediation |
| Overall multidimensional quality mean | 3.701 | -0.736 | -2.723 | [-2.994, -2.439] | Significant negative mediation |
| Behavioral intervention tendency mean | 3.701 | 0.641 | 2.371 | [1.952, 2.794] | Significant positive mediation |

Note: Bootstrap = 2000. The a path represents algorithmic warning predicting perceived AI authorship likelihood; the b path represents perceived AI authorship likelihood predicting the dependent variable. The model simultaneously controlled for visual highlighting and the algorithmic warning × visual highlighting interaction. The mediation analysis was used for mechanism exploration and should not be treated as strict causal mediation evidence.

Appendix Table F3. MANOVA Results for Multidimensional Quality Evaluation

| **Effect** | **Wilks λ** | **F** | **df** | **p** | **Interpretation** |
| --- | --- | --- | --- | --- | --- |
| Algorithmic warning | 0.245 | 213.425 | 3, 208 | <.001 | Significant multivariate effect |
| Visual highlighting | 0.477 | 76.131 | 3, 208 | <.001 | Significant multivariate effect |
| Interaction | 0.947 | 3.896 | 3, 208 | .010 | Significant multivariate effect |

Appendix Table F4. Thematic Coding Results for Open-Ended Revision Suggestions

| **Coding category** | **A Low AI/no highlighting** | **B Low AI/red highlighting** | **C High AI/no highlighting** | **D High AI/red highlighting** | **χ²** | **p** |
| --- | --- | --- | --- | --- | --- | --- |
| Content-development suggestions | 29 (54.7%) | 2 (3.8%) | 9 (16.4%) | 4 (7.4%) | 53.064 | <.001 |
| Language-revision suggestions | 4 (7.5%) | 34 (65.4%) | 2 (3.6%) | 15 (27.8%) | 66.175 | <.001 |
| AI-suspicion suggestions | 0 (0.0%) | 0 (0.0%) | 4 (7.3%) | 4 (7.4%) | 8.007 | .046 |
| Academic-integrity intervention suggestions | 1 (1.9%) | 1 (1.9%) | 14 (25.5%) | 16 (29.6%) | 27.973 | <.001 |

Note: Open-ended responses could receive multiple codes, so category percentages do not sum to 100%. Two coders independently coded all valid Q23 responses using the four non-mutually exclusive categories. Because the categories were not mutually exclusive, inter-coder reliability was assessed separately for each category as a binary present/absent decision. Detailed agreement percentages, Cohen's κ values, and 95% CIs calculated using asymptotic standard errors are reported in Appendix Table F4a. Disagreements were resolved through discussion, and the reconciled codes were used for the χ² analyses reported in this table and for Appendix Figure G3. This analysis remains exploratory and supplementary, but the double-coding procedure supports the reliability of the descriptive coding results.

Appendix Table F4a. Inter-Coder Reliability for Open-Ended Response Coding

| **Category** | **Percent agreement** | **Cohen's κ** | **95% CI** | **Interpretation** |
| --- | --- | --- | --- | --- |
| Content-development suggestions | 91.8% | 0.78 | [0.71, 0.85] | Substantial |
| Language-revision suggestions | 92.4% | 0.82 | [0.76, 0.88] | Almost perfect |
| AI-suspicion suggestions | 97.9% | 0.72 | [0.55, 0.89] | Substantial |
| Academic-integrity intervention suggestions | 93.8% | 0.79 | [0.72, 0.86] | Substantial |

Note: Percent agreement and Cohen's κ were calculated before consensus reconciliation. Because each open-ended response could receive multiple codes, inter-coder reliability was calculated separately for each coding category as a binary present/absent decision. Disagreements were resolved through discussion, and the consensus codes were used for the final thematic frequency and χ² analyses. 95% confidence intervals were calculated using asymptotic standard errors. The wider confidence interval for the AI-suspicion category should be interpreted in light of its low prevalence. Interpretation follows conventional benchmarks in which κ values of approximately 0.61-0.80 indicate substantial agreement and 0.81-1.00 indicate almost perfect agreement.

Appendix Table F5. Randomization-Balance Checks and Q24 Sensitivity Analysis

| **Check** | **Statistic / descriptive result** | **p value** | **Interpretation** |
| --- | --- | --- | --- |
| Discipline (Q26) | χ²(9) = 9.368 | .404 | Approximately balanced across groups. |
| Prior use of AI detection tools (Q28) | χ²(3) = 0.516 | .915 | Approximately balanced across groups. |
| Teaching experience (Q27) | χ²(6) = 8.385; 1-3-year teachers: A = 11, B = 10, C = 4, D = 5 | .211 | Approximately balanced across groups. |
| Acceptance of students' AI-assisted writing (Q29) | F(3, 210) = 4.976; means: A = 3.06, B = 2.96, C = 2.75, D = 2.44 | .002 | Not balanced; reported descriptively only because Q29 was measured after exposure and was not used as a covariate. |
| Q24 sensitivity analysis | Excluding four partially suspicion-aware cases reduced N to 210; results were unchanged. | — | The main conclusions did not depend on these cases. |

Appendix Table F6. Levene's Tests for Homogeneity of Variance

| **Dependent variable** | **Levene's F** | **p** | **Interpretation** |
| --- | --- | --- | --- |
| Overall quality rating (1-10) | 7.052 | <.001 | Homogeneity not met; checked with HC3 robust OLS |
| Course paper score (0-100) | 0.130 | .942 | Homogeneity met |
| Originality mean score | 3.431 | .018 | Homogeneity not met; checked with HC3 robust OLS |
| Language expression mean score | 0.956 | .414 | Homogeneity met |
| Logical structure mean score | 1.566 | .199 | Homogeneity met |
| Overall multidimensional quality mean | 1.911 | .129 | Homogeneity met |
| Behavioral intervention tendency mean | 0.433 | .730 | Homogeneity met |
| Perceived AI authorship likelihood | 2.105 | .101 | Homogeneity met |
| Perceived AI risk | 3.081 | .028 | Homogeneity not met; checked with HC3 robust OLS |
| AI detection report perception mean | 2.692 | .047 | Homogeneity not met; checked with HC3 robust OLS |

Note. Levene's tests use df = 3, 210. Significant p values indicate unequal variances across the four experimental groups. Because several outcomes showed variance heterogeneity, HC3 robust OLS checks were added as supplementary inference rather than replacing the planned ANOVA framework.

Appendix Table F7. HC3 Robust OLS Checks for the 2 × 2 Models

| **Dependent variable** | **Algorithmic warning F**  **(p)** | **Visual cue F**  **(p)** | **Interaction F**  **(p)** | **Interpretation** |
| --- | --- | --- | --- | --- |
| Overall quality rating (1-10) | 278.712 (<.001) | 20.128 (<.001) | 3.119 (.079) | Interaction remained non-significant |
| Course paper score (0-100) | 602.675 (<.001) | 60.866 (<.001) | 32.026 (<.001) | Conclusions unchanged |
| Originality mean score | 374.966 (<.001) | 27.691 (<.001) | 9.739 (.002) | Conclusions unchanged |
| Language expression mean score | 470.622 (<.001) | 86.275 (<.001) | 11.282 (.001) | Conclusions unchanged |
| Logical structure mean score | 229.724 (<.001) | 22.009 (<.001) | 10.365 (.002) | Conclusions unchanged |
| Overall multidimensional quality mean | 373.583 (<.001) | 44.009 (<.001) | 10.947 (.001) | Conclusions unchanged |
| Behavioral intervention tendency mean | 300.501 (<.001) | 30.292 (<.001) | 5.102 (.025) | Interaction remained significant |
| Perceived AI authorship likelihood | 1120.772 (<.001) | 27.719 (<.001) | 1.632 (.203) | Interaction remained non-significant |
| Perceived AI risk | 1113.072 (<.001) | 15.284 (<.001) | 0.000 (.985) | Interaction remained non-significant |
| AI detection report perception mean | 736.115 (<.001) | 100.503 (<.001) | 0.001 (.977) | Interaction remained non-significant |

Note. Models used effect-coded algorithmic warning, effect-coded visual cue, and their interaction. Values are robust F tests with HC3 standard errors; each test has numerator df = 1 and denominator df = 210. The robust checks preserved the same substantive conclusions as the planned ANOVA results.

**Appendix G. Additional Supplementary Tables and Figures**

Appendix Table G1. Data Cleaning and Valid Sample Size

| **Group** | **Raw N** | **Excluded N** | **Valid N** | **Main exclusion criteria** | **Valid sample percentage** |
| --- | --- | --- | --- | --- | --- |
| A Low AI/no highlighting | 58 | 5 | 53 | Clear recall error; straight-lining; contradictory response; attention-check failure. | 91.4% |
| B Low AI/red highlighting | 55 | 3 | 52 | Clear recall error; straight-lining; attention-check failure. | 94.5% |
| C High AI/no highlighting | 59 | 4 | 55 | Contradictory response; clear recall error; abnormal response; attention-check failure. | 93.2% |
| D High AI/red highlighting | 60 | 6 | 54 | Contradictory/abnormal response; straight-lining; clear recall error; attention-check failure. | 90.0% |

Note. Row IDs are anonymized survey IDs used to document reproducible cleaning decisions. In the cleaned analysis workbook, B07 and B46 are already marked as excluded; therefore, the main analytic sample remains N = 214. C45 and D07 were also treated as excluded abnormal/contradictory response patterns rather than retained outliers. For the complete case-by-case exclusion record, see the exclusion column in the supplementary analysis workbook. The sensitivity analysis in Appendix Table F1 re-includes clear recall-check error cases without other exclusion reasons to verify robustness.

Appendix Table G2. Internal Consistency Reliability of Each Scale

| **Scale** | **Number of items** | **Cronbach's α** | **Assessment** | **Mean inter-item correlation r** |
| --- | --- | --- | --- | --- |
| Originality | 3 | 0.981 | High; possible item similarity | 0.949 |
| Language expression | 3 | 0.979 | High; possible item similarity | 0.945 |
| Logical structure | 3 | 0.961 | High; possible item similarity | 0.892 |
| Overall multidimensional quality scale | 9 | 0.988 | High; possible item similarity | 0.921 |
| Behavioral intervention tendency | 3 | 0.817 | Good; moderate-to-high item association | 0.613 |
| AI detection report perception | 5 | 0.972 | High; possible item similarity | 0.904 |


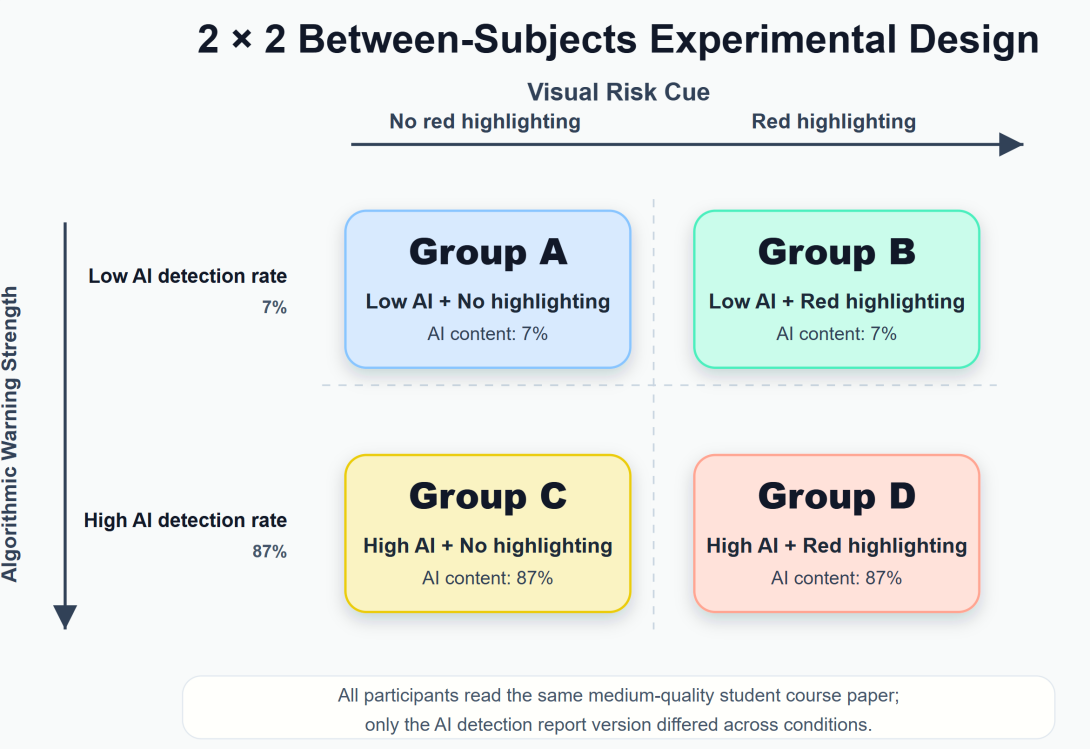
Appendix Figure G1. Experimental Design and Four Conditions


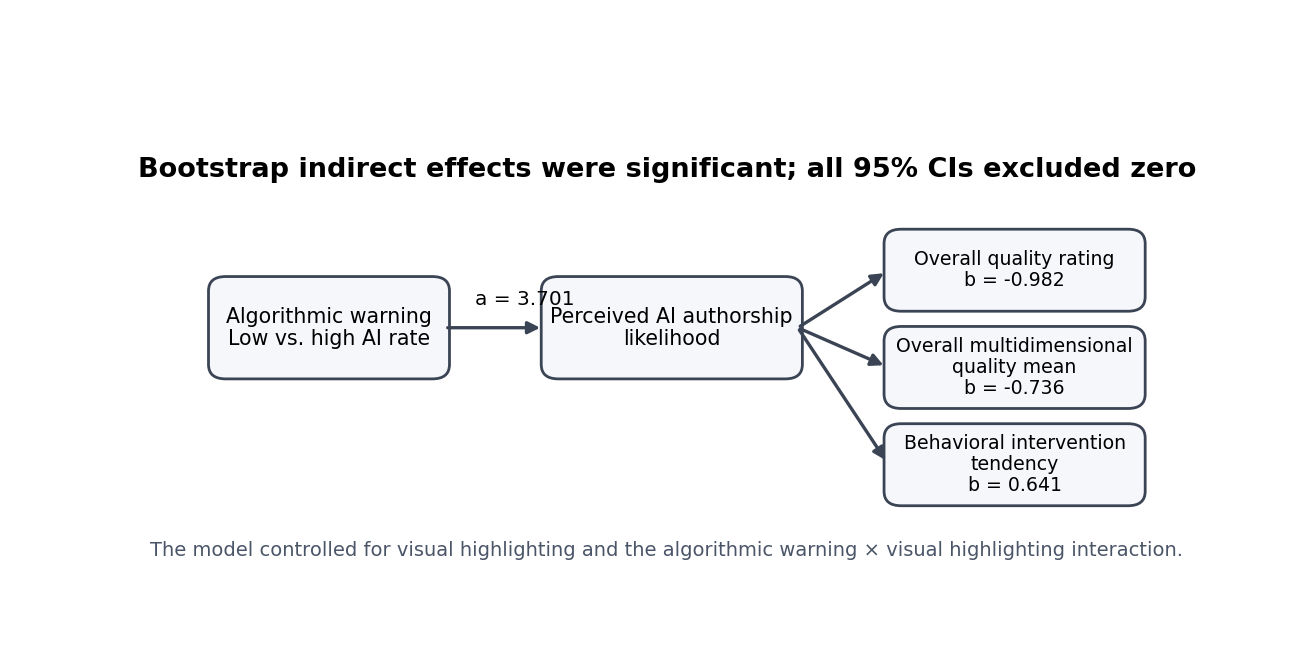


Appendix Figure G2. Mediation Mechanism of Perceived AI Authorship Likelihood


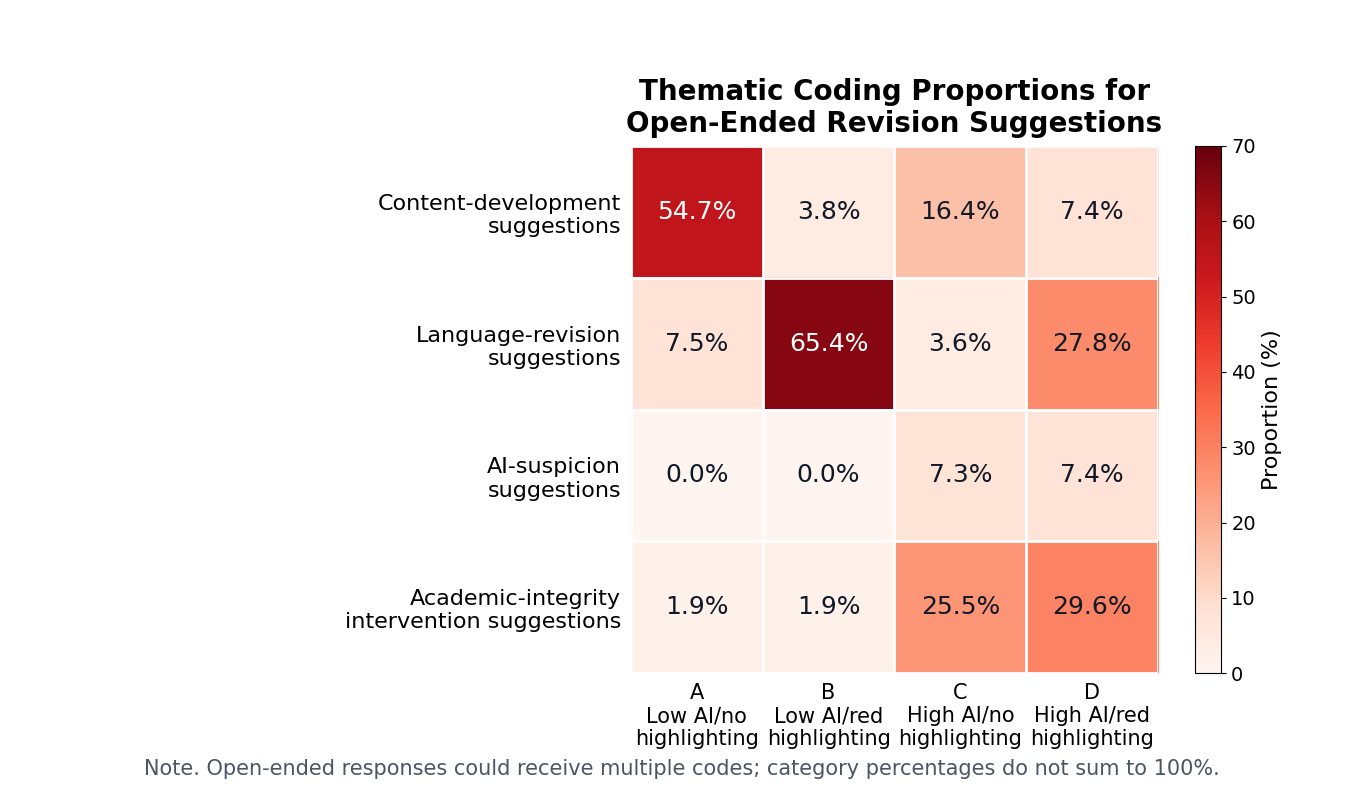


Appendix Figure G3. Thematic Coding Proportions for Open-Ended Revision Suggestions
